# Assessment of the oxidative damage and apoptotic pathway related to furan cytotoxicity in cultured mouse Leydig cells

**DOI:** 10.1093/toxres/tfad025

**Published:** 2023-04-20

**Authors:** Yasemin Aydin, Buse Yilmaz, Yasemin U Dikbasan, Banu Orta-Yilmaz

**Affiliations:** Department of Biology, Faculty of Science, Istanbul University, Istanbul 34116, Turkey; Institute of Graduate Studies in Science and Engineering, Department of Biology, Istanbul University, Istanbul 34116, Turkey; Institute of Graduate Studies in Science and Engineering, Department of Biology, Istanbul University, Istanbul 34116, Turkey; Department of Biology, Faculty of Science, Istanbul University, Istanbul 34116, Turkey

**Keywords:** furan, Leydig cells, cytotoxicity, apoptosis, oxidative stress, antioxidant system

## Abstract

Research on heat-induced food contamination is being given more attention as a result of the health risks that have been publicly revealed in recent years. Furan is known as a colorless, combustible, heterocyclic aromatic organic molecule and is formed when food products are processed and stored. It has been established that furan, which is inevitably ingested, has a deleterious impact on human health and causes toxicity. Furan is known to have adverse effects on the immune system, neurological system, skin, liver, kidney, and fat tissue. Infertility caused by furan is a result of its damaging effects on several tissues and organs as well as the reproductive system. Although studies on the adverse effects of furan on the male reproductive system have been performed, there is no study revealing apoptosis in Leydig cells at the gene level. In this study, TM3 mouse Leydig cells were exposed to 250- and 2,500-μM concentrations of furan for 24 h. The findings demonstrated that furan decreased cell viability and antioxidant enzyme activity while increasing lipid peroxidation, reactive oxygen species, and apoptotic cell rates. Furan also increased the expression of the important apoptotic genes *Casp3* and *Trp53* while decreasing the expression of another pro-apoptotic gene, *Bcl2*, and antioxidant genes *Sod1*, *Gpx1*, and *Cat*. In conclusion, these results imply that furan may cause loss of cell function in mouse Leydig cells responsible for testosterone biosynthesis by impairing the efficiency of the antioxidant system, possibly by inducing cytotoxicity, oxidative stress, and apoptosis.

## Introduction

Furan is one of the thermal-sourced food contaminants that are commonly consumed in daily diets and to which most people are exposed due to their presence in numerous food products. Furan develops naturally in foods after thermal processing including baking, roasting, pasteurization, cooking, and sterilizing.^1^^,^^2^ Furan is formed during the Maillard reaction, also known as the non-enzymatic browning reaction. Thermal degradation of carbohydrates such as glucose, lactose and fructose are the primary source of furan.^4^ Furan is also found in environment as a component of smoke and exhaust fumes.^5^ The level of furan in various foods has been determined by food analyses conducted by several health authorities, including the US Food and Drug Administration (FDA), the Ministry of Health Canada, the European Food Safety Authority (EFSA), and the Swiss Public Health Agency. In addition, furan has been found in a variety of foods, including roasted coffee, baby food, cereal products, beverages, canned vegetables, cooked and canned meats, beer, and wheat breads.^1^^,^^6^ According to EFSA studies on furan concentrations in heat-treated food products, brew coffee is the most prevalent source of furan exposure for adults. In the same study, while the furan concentration in brewed and roasted coffee was 42 and 3,660 ng/g, it has been shown that it is between .2 and 3.2 ng/g in baby food, 22–24 ng/g in bakery products, and 13–17 ng/g in meat products.^2^^,^^7^

Many studies have reported that furan has negative effects on the biological systems of humans and animals. According to previous studies, furan causes a wide range of toxicity, including hepatotoxicity,^8^ carcinogenicity,^9^ lung, renal,^10^ and ovarian damage.^11^^,^^12^ Moreover, many in vivo studies on the male reproductive system have indicated that furan causes impairments in the testis, epididymis and prostate gland,^3^^,^^12^^,^^13^^,^^14^ spermatogenesis and steroid production,^15^ as well as lower levels of luteinizing hormone, follicle stimulating hormone, and testosterone.^3^^,^^16^^,^^17^ Several studies have shown that furan significantly increases apoptotic gene expression (*Caspase 3*, *Caspase 9*)^18^^,^^19^ and the formation of apoptotic cells in the rat testis.^3^^,^^12^ It is critical to investigate the effects of furan on male reproductive system due to the complex structure, physiology, and regulatory significance of the male reproductive system. There is in vivo research on the toxicity of furan on the male reproductive system, however no in vitro gene-level studies showing how furan affects apoptosis in Leydig cells. In this study, Leydig cells, which are the main cells that produce testosterone in the male reproductive system, were utilized as model cells to determine the effect of furan on male infertility. The purpose of this study was to investigate how furan exposure affected the antioxidant system and apoptosis in Leydig cells.

## Material and method

### Chemicals and reagents

Furan was obtained from Sigma Aldrich (cat. no. 110-00-9). Dulbecco’s modified Eagle’s medium/F12, horse serum, fetal bovine serum, Penicillin-Streptomycin (cat. no. 450-200-EL) and trypsin were purchased from Wisent Bioproducts (Quebec, Canada). Other chemicals used in experiments were purchased from Sigma Aldrich (St. Louis, MO) and Merck (Darmstadt, Germany).

### Cell culture and treatment

Mouse TM3 Leydig cells (ATCC, Manassas, VA, USA), non-tumorigenic cells, were cultured in DMEM/F12 with 5% horse serum and 2.5% fetal bovine serum at 37°C under a humidified atmosphere of 5% CO_2_. Cells were maintained as a monolayer culture in a 75 cm^2^ culture flask. Growth media of the cultured cells was replaced three to four times weekly and the cells were sub-cultured when confluence reached 70%. For the experiments, the cells were seeded in 6–24- and 96-multiwell plates at appropriate densities and allowed to adhere overnight. Furan was dissolved in 99% ethanol, and it was diluted with DMEM/F12 to obtain a 3 mM furan stock solution with .1% ethanol. Then, the stock concentration was further diluted with DMEM/F12 to working concentrations of 250 (no effects of cell viability) and 2,500 μM (decrease cell viability ratio to 75%) furan. All experiments were carried out in duplicate or triple biological replicates, and each experiment was independently repeated at least three times.

### Cell viability assay

The effects of furan on Leydig cells viability were assessed using the colorimetric 3-(4,5 dimethylthiazol-2-yl)-2,5-diphenyl tetrazolium bromide (MTT) assay, according to the manufacturer’s instructions (Roche Diagnostics GmbH, Mannheim, Germany). The MTT assay, which is used to evaluate mitochondrial dehydrogenase activity in living cells, is based on the capacity of metabolically active cells to reduce MTT to purple formazan crystals. Leydig cells were seeded in a 96 well-plate at a density of 5 × 10^3^ cells per well. After the cells were adherent to the culture plates, they were treated to various concentrations of furan (250, 500, 1,000, 1,500, 2,000, 2,500 μM) for 24 h prepared in medium containing 1% horse serum. After 24 h of treatment, furan-containing experiment media in the culture-plate was removed and cells were incubated with 10-μl MTT I solution for 4 h avoiding light. Formed formazan crystals by metabolically active cells were dissolved by adding 100 μl/well of MTT II (solution sodium dodecyl sulphate) and the samples were incubated overnight in a CO_2_ incubator. Afterwards, the absorbance of the mixture was measured at 540 nm using a microplate spectrophotometer (Multiskan Spektrum, Thermo Fisher Scientific Inc., Waltham, MA, USA). Viability ratios of cells were calculated by assuming the control group to be 100%.^20^

### Detection of membrane integrity with lactate dehydrogenase assay

Lactate dehydrogenase (LDH), a cytoplasmic enzyme, is considered a cytotoxicity marker. When a cell dies or the cell membrane is damaged, LDH is released into the extracellular area. Thus, cell membrane integrity damaged by the test substance is analyzed by measuring the LDH activity released into the culture media. The quantity of LDH enzyme was obtained using the micro plate-based Cytotoxicity Detection Kit (Roche Molecular Biochemicals, Mannheim, Germany) according to the manufacturer’s protocol. The cells were cultured in 96-well plates (1 × 10^4^ cells/well) and exposed to furan at concentrations of 250 and 2,500 μM for 24 h. After the exposure period had ended, 100 μl of LDH assay mixture was added to culture supernatants and incubated for 30 min. LDH release levels were determined by measuring absorbance with a microplate reader at 492-nm wavelength. LDH leakage in control cells cultured with furan-free medium was assumed to be 100% and cytotoxicity percentages of experimental cells were calculated compared with control cells.

### Biochemical analysis

TM3 Leydig cells (1 × 10^6^ cells/well) were seeded in culture plates to determine biochemical parameters such as lipid peroxidation, reactive oxygen species, and antioxidant enzyme levels. After the furan exposure for 24 h the cells were incubated with trypsin for 3 min and transferred into Tris–HCl buffer at a pH of 7.2. Then, the collected cells were sonicated with an ultrasonicator (Bandelin Electronic, Berlin, Germany) to disrupt the cell membranes. The cell suspensions were centrifuged at 14,000 g. After the centrifugation, the supernatants were preserved at −86°C for biochemical analysis.

### Determination of oxidative stress indicators

Lipid peroxidation was measured according to the reaction product malondialdehyde (MDA) content by using the method of Heath and Packer’s.^21^ This method is based on the principle of measuring the pink chromogen formed by the reaction with thiobarbituric acid (TBA) at 532–535 nm to measure the MDA level. The reaction mixture containing .5% ΤΒΑ, diluted with 20% trichloroacetic acid (TCA) was added to .1 ml of the supernatant. Then the mixture was incubated in a 100°C water bath for one hour. After the incubation, the mixture was centrifuged at 10,000 g for 5 min. Then the absorbance of the supernatant was measured at 532 and 600 nm in the spectrophotometer and the results were recorded.

Levels of hydroxyl radical (OH•) were determined according to the method of Puntarulo and Cederbaum.^22^ This method is based on the principle of detecting the formaldehyde that the hydroxyl radical produces when it interacts with iron complexes. In brief, the cell extract was mixed with sodium phosphate buffer (1 M), magnesium chloride (.1 M), sodium azide (10 mM), DMSO (4 mM), and nicotinamide adenine dinucleotide phosphate (NADPH) (4 mM). After a 10-min incubation period at 37°C, the samples were treated with a 10% TCA solution to terminate the reaction and boiled for 30 min in water bath. Then, using a spectrophotometer set to 570 nm, the absorbance of the samples associated with the hydroxyl radical level was determined and expressed as mmol/min per mg protein.

For the assessment of hydrogen peroxide (H_2_O_2_), the method described by Holland and Storey^23^ was used. In order to quantify H_2_O_2_, this technique employs an absorption at 550 nm that results from the oxidation of acidified ferrocytochrome c. The cell extract was mixed with 20-mM Tris–HCl, 113-mM potassium chloride, .4-mM ethylenediamine tetra acetic acid, 15-mM KH_2_PO_4_, and 98-M ferrocytochrome c. The H_2_O_2_ level of the sample was then quantified as mol/min per mg protein using spectrophotometric readings at 550 nm.

### Evaluation of activity of antioxidant enzymes

The activity of superoxide dismutase (SOD) was evaluated using the Marklund and Marklund’s method.^24^ The concept behind this technique is inhibition of the pyrogallol auto-oxidation by the SOD enzyme. Briefly, the reaction mixture was prepared from .05-ml cell extract, 1.4-ml Tris–HCl buffer (pH 8.2) and .05-ml of 2 mM pyrogallol. At 420 nm, the variation in spectrophotometric value was recorded for 3 min with a 30-s interval. The enzyme activity is expressed as units of SOD per milligram of protein.

Catalase (CAT) activity was measured according to Sinha’s method.^25^ The aim of this technique is to reveal that dichromate in acetic acid is converted into chromic acetate when heated in the presence of H_2_O_2_. To start the reaction, .1 mL of cell extract was added to the mixture of .4 mL of .2-M H_2_O_2_ and .5 ml of sodium phosphate buffer. Then, 2 ml of dichromate/acetic acid reagent was added to the mixture to block the reaction. The final solution was incubated in a boiling water bath for 10 min. After cooling, the absorbance of the chromic acetate was recorded with a spectrophotometer at a wavelength of 560 nm.

Glutathione peroxidase (GPx) activity was assessed using the Hafeman et al. technique.^26^ When reduced glutathione is present, GPx catalyzes the hydrolysis of H_2_O_2_, which produces oxidized glutathione and water. The test mixture, which contained .15 ml of cell extract, .5 ml of sodium phosphate buffer (.4 M), .25 ml of sodium azide (10 ml), .5 ml of reduced GSH (4 ml), and .6 ml of distilled water, was preheated to 37°C and incubated for 5 min. After that, the mixture received .5 ml of 1.25 mM H_2_O_2_ before the tubes underwent a 3 min incubation period at 37°C. To terminate the reaction, 1 ml of 10% TCA was added to the mixture. After centrifugation, 4 ml of Na_2_HPO_4_ solution and 1 ml of DTNB reagent were added to the supernatant. The absorbance values in the reaction mixture were then examined at 412 nm.

The glutathione s-transferase (GST) method developed by Habig et al. was used to evaluate the GSH-S-transferase activity.^27^ The reaction between GSH and 1-chloro-2,4-dinitrobenzene (CDNB) can be catalyzed by GST. .02 ml of cell extract, 9.8 ml of potassium phosphate buffer, .1 ml of CDNB, .1 ml of reduced GSH, and .1 ml of CDNB were mixed together in test tubes. Following mixing, the solution’s absorbance was measured at 340 nm for 5 min at 60-s intervals.

### Apoptosis measurement by double fluorescent staining

The DNA-binding dyes Hoechst 33342 (HO342) and propidium iodide (PI) were used to label the cells and measure the degree of apoptosis. This method is based on counting and scoring of live, apoptotic, and dead cells. HO342, a blue fluorescent dye, stains the condensed chromatin in apoptotic cells, whereas PI, a red fluorescent dye, stains dead cells. In a summary, 24 well plates containing 1 x 10^4^ Leydig cells per well were cultured for an overnight period before being exposed to furan at different doses for 24 h. After the exposure, a phosphate buffered saline (PBS) solution was used to wash the cells. After that, each well received .2 ml of the test solution, which also contained 20 μl of PI (1 mg/ml), 20 μl of HO342 (1 mg/ml), and 3,960 μl of PBS. This solution was then incubated for 15 to 30 min at 37°C. After the incubation, the cells were washed with PBS twice. After the staining, randomly chosen areas were viewed with a UV filter using an Olympus IX71 fluorescent attachment microscope and were then photographed in a series at regular intervals using an Olympus DP72 video camera (Tokyo, Japan). Ultimately, 1,000 cells were counted over a series of photographs for each experimental group to determine the proportion of live, apoptotic, and dead cells.

### RNA extraction and real-time polymerase chain reaction (real time PCR)

Genes for apoptotic and antioxidant enzymes (*Bcl2, Casp3, Trp53, Sod1, Cat, Gpx1*) were examined using real-time polymerase chain reaction (PCR) analysis. Total RNA was obtained from furan treated samples using RNeasy Mini Kits (Qiagene, Hilden, Germany) in accordance with the manufacturer’s instructions after 24 h of furan exposure (250 and 2,500 μM) to TM3 Leydig cell cultures in six-well plates with 1 x 10^6^ cells per well. Using the ND2000c NanoDrop device, the nucleic acid content (ng/l) and purity levels (A260/230) in the acquired RNA samples were assessed (Thermo Fisher Scientific). The first strand complementary DNA was synthesized from total RNA using Turbo 1 reverse transcriptase (Biomatic, Ontario, Canada) with oligo-dT primers. According to the manufacturer’s instructions, random primers were initially designed (Integrated DNA Technologies, Leuven, Belgium). The primers used were as follows: *Sod1* (forward: 5′-GTGATTGGGATTGCGCAGTA-3′; reverse: 5′-TGGTTTGAGGGTAGCAGATGAGT-3′), *Cat* (forward: 5′-TTGGCAAACTGGTTTTAAACAAAAATCC-3′; reverse: 5′-GACATTGTCTTCATTAGCACTGTTGA-3′), *Gpx1* (forward: 5′-CGCTTTCGTACCATCGACATC-3′; reverse: 5′-GGGCCGCCTTAGGAGTTG-3′), *Bcl2* (forward: 5′-GACTGAGTACCTGAACCGG-3′; reverse: 5′-ATAGTTCCACAAAGGCATCC-3′), *Casp3* (forward: 5′-AATGGATTATCCTGAAATGGGC-3′; reverse: 5′-GAGCGAGATGACATTCCAG-3′), *Trp53* (forward: 5′-AGAGACCGCCGTACAGAAGA-3′; reverse: 5′-GCATGGGCATCCTTTAACTC-3′), β*-actin* (forward: 5′-CGTTGACATCCGTAAAGAC-3′; reverse: 5′-TGGAAGGTGGACAGTGAG-3′). The samples were heated to 85°C for 5 min to terminate the reaction after 10 min of reverse transcription at 25°C, followed by 50 min at 42°C of incubation. For each sample, reverse transcription reaction was performed in triplicate in a thermal cycler. Real-time PCR experiments were carried out on a LightCycler 480 RTPCR system (Roche) using a LightCycler 480 SYBR Green 1 Master (Roche) kit in accordance with the manufacturer’s instructions to quantify the targeted gene expression. The PCR reactions were performed as follows: pre-denaturation at 95°C for 5 min, 45 cycles of denaturation at 95°C for 10 s, annealing at 57°C for Tm degrees, elongation at 72°C for 25 s. To normalize the data, the housekeeping gene β*-actin* was used as an internal control. The 2 ^−ΔΔCt^ method was used to calculate the relative expression ratio.^28^

### Statistics

Data were statistically analyzed by Tukey’s multiple comparisons and a one-way ANOVA using GraphPad Prism 6 Software (GraphPad Prism Software, San Diego, California, USA). The normality of the data distribution was evaluated using Shapiro–Wilk to determine the statistical significance of the RT-PCR results. All results were expressed as the mean ± standard deviation. The values of *P* < .05 were regarded as statistically significant, whereas the values of *P* < .01 were considered extremely significant.

## Results

### Cytotoxicity of furan on Leydig cells

**Fig. 1:**
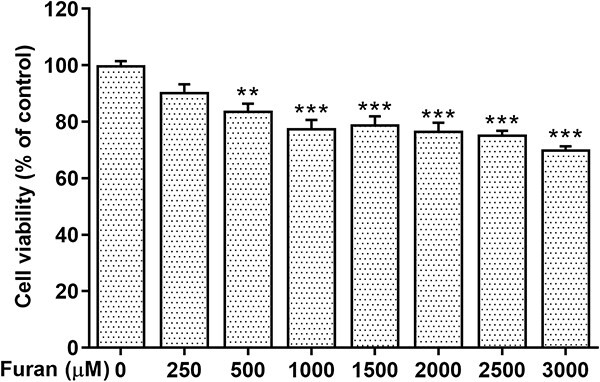
Concentration-dependent effects of furan on cell viability of TM3 Leydig cells for 24 h in vitro. Each bar represents the mean (±SEM) of three independent experiments carried out in triplicates. ^*^^*^P < .01, ^*^^*^^*^P < .001.

**Fig. 2:**
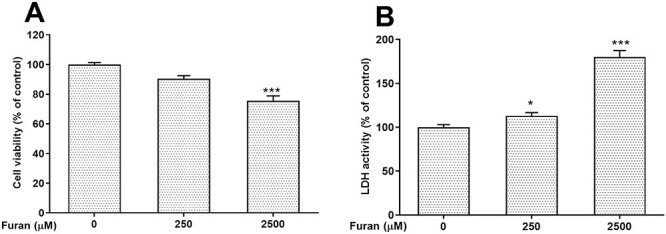
Concentration-dependent cytotoxicity of furan on TM3 Leydig cells. (A) Cell viability was measured by MTT assay in TM3 Leydig cells exposed to two different concentrations of furan. (B) The effects of furan on cell membrane damage by lactate dehydrogenase activity in TM3 Leydig cells for 24 h. all data are presented as the means (± SEM) of three independent experiments carried out in triplicates. ^*^P < .05, ^*^^*^^*^P < .001.

The cell viability of TM3 cells induced by furan was investigated using the MTT test at exposure concentrations ranging from 250 to 3,000 μM for 24 h. As described in Fig. 1, with the increase of furan concentration, the cell viability rate of the experimental groups exposed to 500–3,000 μM furan concentration decreased significantly compared with the control group (^*^^*^P < .01, ^*^^*^^*^P < .001). According to the calculated MTT results, 250-μM furan concentration decreased cell viability to 9.33%, whereas 2,500-μM furan concentration decreased to 75.55% (Fig. 2A).

LDH enzyme was quantified by measuring the rate of decrease in nicotinamide adenine dinucleotide phosphate in cells, and the degree of plasma membrane damage due to furan concentration in cells was revealed. According to the findings, the LDH activity of both experimental groups (250- and 2,500-μM furan) increased significantly as compared with the un-treated group (^*^P < .05, ^*^^*^^*^P < .001) (Fig. 2B).

### Increases in biomarkers of oxidative stress in Leydig cells exposed to furan

In this study, MDA, OH•, and H_2_O_2_ levels were analyzed in furan exposed Leydig cells to assess the degree of oxidative stress. As shown in Table 2, furan exposure (250- and 2,500-μM furan) consistently and significantly increased the MDA levels in a concentration-dependent manner (^*^P < .05). These findings demonstrate that furan activates oxidative stress-inducing lipid peroxidation in Leydig cells. Moreover, the Leydig cells treated with furan showed considerable changes in the levels of OH• and H_2_O_2_ (Table 1). In comparison with the control groups, a prominent increase in OH• and H_2_O_2_ levels was observed in both the furan (250- and 2,500-μM furan) exposed groups (^*^P < .05).

**Table 1:** Concentration dependent effects of furan on oxidative stress parameters in TM3 Leydig cells.

Groups	MDA (μmol/mg protein)	OH^•^ (μmol/mg protein)	H₂O₂ (μmol/mg protein)	
Control	1.04 ± .1	2.63 ± .5	3.16 ± .4
Furan (250 μM)	3.19 ± .3^a^	4.45 ± .3^a^	5.24 ± .3^a^
Furan (2,500 μM)	4.02 ± .7^a^^,^^b^	6.06 ± .8^a^^,^^b^	1.28 ± .9^a^^,^^b^

Each value denotes mean ± SEM of five independent experiments carried out in triplicates. Significance at *P < .*05*.*

^a^Significant *P*-value versus control group.

^b^Significant *P*-value between furan (250 μM) and furan (2,500 μM) groups.

### Deteriorations in antioxidant system parameters in Leydig cells exposed to furan

**Fig. 3:**
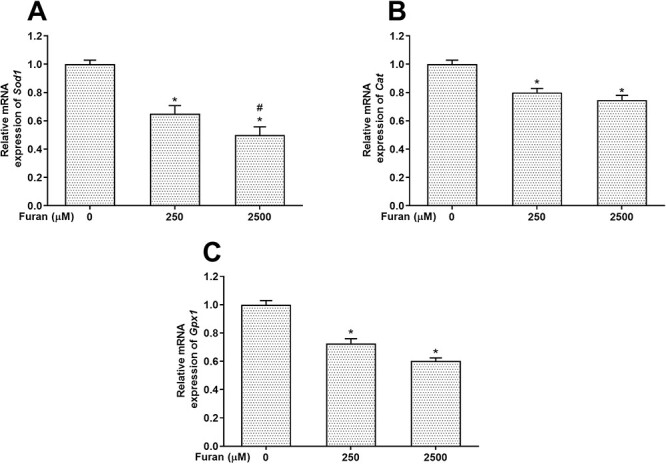
The effects of furan on the mRNA expression levels of *Sod1* (a), *cat* (B), and *Gpx1* (C) in TM3 Leydig cells. All data are presented as the means (± SEM) of three independent experiments carried out in duplicates. ^*^P < .05 compared with control and #*P* < .05 compared with 250-μM furan concentration.

The activity levels of various antioxidant enzymes (SOD, CAT, GPx, GST) were measured to evaluate oxidative damage due to furan exposure. The changes in SOD, CAT, GPx, and GST enzyme activities of cultivated Leydig cells exposed to two different concentrations of furan were given in Table 2. According to the findings, a significant decrease was revealed in the SOD and CAT enzyme levels of the cells exposed to both 250- and 2,500-μM furan concentrations for 24 h compared with the control group (^*^P < .05). GPx enzyme activity indicated a significant decrease in all experimental groups, especially those exposed to the highest concentration of furan (2,500 μM) (^*^P < .05). Compared with the control group, GST enzyme activity was markedly reduced in both experimental groups after 24 h of furan exposure in a concentration-dependent manner (^*^P < .05). In our study, antioxidant system parameters were also evaluated in terms of gene expression levels. When the expression levels of *Sod1* (Fig. 3A), *Cat* (Fig. 3B), and *Gpx1* (Fig. 3C) genes were examined, it was found that they were significantly downregulated in the furan-exposed groups, similar to the enzyme levels (^*^P < .05).

**Table 2:** Concentration dependent effects of furan on antioxidant enzyme levels in TM3 Leydig cells.

Groups	SOD (U/mg protein)	CAT (nmol of H_2_O_2_ consumed/min/mg protein)	GPx (nmol of glutathione consumed/mg protein)	GST (nmol of CDNB-GSH complex formed/mg protein)
Control	54.20 ± 2.1	3.10 ± .9	5.11 ± .5	0.46 ± .0
Furan (250 μM)	38.59 ± 1.8^a^	2.40 ± .3^a^	3.80^a^±.3	0.26 ± .0^a^
Furan (2,500 μM)	24.40 ± 1.9^a^^,^^b^	1.91 ± .8^a^^,^^b^	2.56 ± .1^a^^,^^b^	0.19 ± .0^a^^,^^b^

Each value denotes mean ± SEM of five independent experiments carried out in triplicates. Significance at *P* < .05*.*

^a^Significant *P*-value versus control group.

^b^Significant *P*-value between furan (250 μM) and furan (2500 μM) groups.

### Changes observed in the apoptotic pathway in Leydig cells exposed to furan

**Fig. 4:**
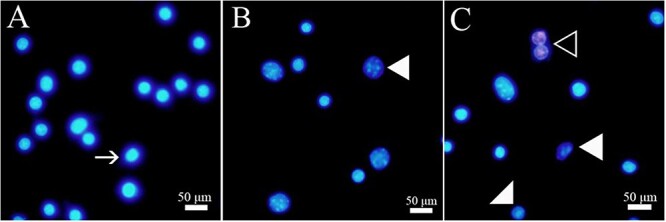
Concentration-dependent effects of furan on apoptosis rate in TM3 Leydig cells. (A) Control, (B) 250-μM furan, (C) 2,500-μM furan. ←, viable cell; ▼, apoptotic cell; <, dead cell. Scale bar, 50 μm.

**Fig. 5:**
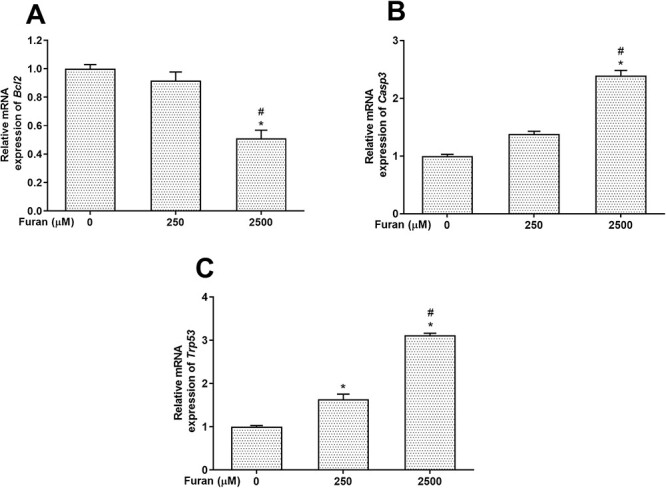
The effects of furan on the mRNA expression levels of apoptotic genes *Bcl2* (A), *Casp3* (B), and *Trp53* (C) in TM3 Leydig cells. All data are presented as the means (± SEM) of three independent experiments carried out in duplicates. ^*^P < .05 compared with control and #*P* < .05 compared with 250-μM furan concentration.

Double fluorescence staining using PI and HO342 was performed to analyze the cell apoptosis (Fig. 4). The results showed that there was a concentration-response relationship between furan concentrations and the rate of apoptotic cells (Table 3). Accordingly, furan-exposed cells showed a considerably higher frequency of apoptosis compared with the control groups. Also, the number of dead cells was significantly increased depending on furan concentrations (^*^P < .05). When the expression levels of *Bcl2*, *Casp3*, and *Trp53* genes, which play a critical role in the apoptotic pathway, were examined, no change was observed in levels of *Bcl2* gene expression at 250-μM furan concentration, but a significant increase was found at 2,500 μM furan concentration (Fig. 5A). When *Casp3* gene expression levels were compared in Leydig cells, no significant change was observed at low concentration of furan, whereas transcription of the *Casp3* gene was significantly increased at high concentration of furan (Fig. 5B). Compared with the control group, *Trp53* expression levels were markedly upregulated in both experimental groups after 24 h of furan exposure in a concentration-dependent manner (Fig. 5C).

**Table 3:** Concentration dependent effects of furan on viable, apoptotic, and dead cells in TM3 Leydig cells.

Groups	Viable cells	Apoptotic cells	Dead cells	
Control	98.4 ± 3.1	1.3 ± .9	0.3 ± .0
Furan (250 μM)	92.4 ± 4.2	7.1 ± 1.2^a^	0.5 ± .0
Furan (2,500 μM)	80.09 ± 2.8^a^^,^^b^	15.2 ± 1.7^a^^,^^b^	3.9 ± .9^a^^,^^b^

Each value denotes mean ± SEM of five independent experiments carried out in triplicates. Significance at *P* < .05*.*

^a^Significant *P*-value versus control group.

^b^Significant *P*-value between furan (250 μM) and furan (2,500 μM) groups.

## Discussion

Although it is known that furan causes toxicity in numerous organs, there have been few investigations at the cell level that will provide a complete understanding of the damage in the male reproductive system.^10^^,^^29^^,^^30^^,^^31^^,^^32^^,^^33^ There are in vivo studies that have documented the damage furan causes to the male reproductive system in the literature; however, there are no in vitro studies demonstrating the furan causes to Leydig cells in the testis.^12^^,^^13^^,^^14^ There are a very limited number of studies demonstrating the effects of furan on cell viability and number at the cellular level.^3^^,^^32^ This is the first study to investigate the cell viability of furan in male reproductive system cells. The results of the current in vitro study with Leydig cells, which showed that 2,500 μM furan significantly reduced cell viability, are consistent with the small number of studies described above in which significant reductions in cell viability as a result of furan administration have been observed.

LDH is a cytoplasmic enzyme found in all cells. When the plasma membrane is damaged, it is quickly released from the cell. Cell cytotoxicity is indicated by the LDH enzyme leaking from the intracellular environment to the extracellular space. The LDH enzyme activity in the cell increases in proportion to the number of dead or damaged plasma membrane of cells.^34^ According to numerous studies, exposure to furan significantly raises the serum LDH levels in mice and rats.^10^^,^^12^^,^^31^ In accordance with these studies, it was revealed that applying furan concentrations (250–2,500 μM) to Leydig cells for 24 h greatly increased LDH activity and resulted in cytotoxicity.

Reactive oxygen species (ROS) formation in cells causes mutations in protein and DNA, lipid peroxidation in membranes, apoptosis, and damage to cellular structures.^35^ Testicular tissue, which is the main component of the male reproductive system and has a low oxygen content due to its limited vascularity, is more susceptible to oxidative stress. Furthermore, it is well-known that oxidative stress in the testis is detrimental to spermatogenesis and steroidogenesis. Despite having low oxygen levels, the testes are vulnerable to oxidative stress because they have high levels of unsaturated fatty acids and ROS-producing mechanisms. Lipid peroxidation, which is a process that occurs as a result of a chain reaction between unsaturated fatty acids in cell membrane lipids and ROS, causes functional disorders by disrupting the cell membrane structure under stress conditions.^36^ Furan causes lipid peroxidation by raising MDA levels in testicular tissue, according to numerous in vivo studies.^3^^,^^12^^,^^16^ An in vivo investigation with albino rats found that exposure to furan substantially increased the levels of ROS and MDA.^31^ Another in vivo study with mice showed a significant increase in the amount of ROS and MDA in the furan-treated groups.^10^ Another investigation using mice found that the toxicity of the furan compound resulted in a significant increase in MDA levels.^33^ According to a study with male rats, ROS activity increased significantly depending on the furan concentration.^14^ In vivo research using rat testis revealed that enzyme activity reduced, and MDA levels increased in groups treated with furan.^30^ Although there are in vivo studies showing excessive ROS production, lipid peroxidation, and MDA increase in furan, there are no in vitro studies on the male reproductive system. This in vitro study has demonstrated that furan causes lipid peroxidation by increasing the levels of hydroxyl radical and hydrogen peroxide in Leydig cells.

Under typical conditions, the cell defense system utilizes both enzymatic and non-enzymatic antioxidants (such as glutathione) to maintain the intracellular redox balance.^37^ Intracellular enzyme levels of catalase, superoxide dismutase, glutathione peroxidase, and glutathione rise following oxidative stress in biological systems, and the antioxidant defense mechanism is triggered.^38^ Studies show that furan suppresses the antioxidant defense system.^10^^,^^16^^,^^14^^,^^30^ Studies conducted in vivo have demonstrated that furan toxicity significantly reduces the levels of the CAT, SOD, GPx, and GST enzymes in lung and testicular tissues.^13^^,^^14^^,^^16^^,^^30^ The levels of antioxidant enzyme expression at the gene level were examined for the first time in this study. The results showed that both furan concentrations applied to Leydig cells drastically decreased enzyme activity and gene expression levels, resulting in impairment of the antioxidant defense system.

Cell homeostasis and energy-dependent biochemical pathways are involved in the intricate process of apoptosis, also referred to as programmed cell death.^39^ Oxidative stress, which develops as a result of exposure to substances like environmental contaminants and hormone-disrupting chemicals, causes cells to undergo apoptosis. Oxidative stress damages the mitochondrial membrane, which results in apoptosis.^40^ Numerous studies claim that various apoptotic processes are used by furan to exert its effects.^12^^,^^19^^,^^41^ According to a study, furan causes apoptosis by increasing caspase 3 activity.^12^ In a different study with furan, it was demonstrated that exposure to the chemical increased the activity of the apoptosis-related genes *caspase 3* and *caspase 9*, respectively.^18^ The expression levels of the furan apoptosis-related genes *caspase 3* and *bcl-2* were considerably altered, according to Chen et al.^19^ The findings of this study on TM3 Leydig cells indicate that furan causes apoptosis by increasing the number of apoptotic cells in Leydig cells, which is consistent with studies conducted in vivo. Additionally, our data showed that exposure to furan significantly increased the expression levels of the genes *Trp53* and *Casp3*, which are crucial in the apoptotic pathway, while considerably decreasing the expression levels of *Bcl2*.

## Conclusion

In conclusion, it was determined in our study that furan decreased cell viability and increased the amounts of the LDH, ROS levels, and lipid peroxidation, which are toxicity markers. Furan has also been shown to cause oxidative damage in Leydig cells by decreasing the activity and gene expression levels of enzymes involved in the intracellular antioxidant defense system, CAT, SOD, and GPx. Additionally, it was demonstrated that furan promoted apoptosis in Leydig cells, both by double immune fluorescence staining and by determining the expression levels of apoptotic genes. Consequently, it is considered that identifying the negative consequences of furan exposure in Leydig cells may contribute to the control and regulation of self-released furan levels during the processing of food products.

## Funding

This study was supported by Istanbul University Scientific Research Projects (Project No. 33276 and 32762).

## Conflict of interest statement

None declared.

## Author contribution

Yasemin Aydin: Project administration, Funding acquisition, Conceptualization, Methodology, Investigation, Analysis experimental results, Visualization, Writing—original draft, Writing—review and editing. Banu Orta-Yilmaz: Project administration, Funding acquisition, Conceptualization, Methodology, Investigation, Analysis experimental results, Visualization, Writing—review and editing. Buse Yilmaz: Investigation, Preparation of materials of reagents, Reference checking. Yasemin Ülkü Dikbasan: Investigation, Preparation of materials of reagents, Reference checking.

## Data availability statement

All data are available from the corresponding author upon reasonable request.
